# Large ^31^P-NMR enhancements in liquid state dynamic nuclear polarization through radical/target molecule non-covalent interaction†

**DOI:** 10.1039/d2cp04092a

**Published:** 2022-12-13

**Authors:** Maik Reinhard, Marcel Levien, Marina Bennati, Tomas Orlando

**Affiliations:** ESR Spectroscopy Group, Max Planck Institute for Multidisciplinary Sciences, Am Faßberg 11 Göttingen Germany tomas.orlando@mpinat.mpg.de; Department of Chemistry, Georg-August-University, Tammannstraße 4 Göttingen Germany

## Abstract

Dynamic nuclear polarization (DNP) is a method to enhance the low sensitivity of nuclear magnetic resonance (NMR) *via* spin polarization transfer from electron spins to nuclear spins. In the liquid state, this process is mediated by fast modulations of the electron-nuclear hyperfine coupling and its efficiency depends strongly on the applied magnetic field. A peculiar case study is triphenylphosphine (PPh_3_) dissolved in benzene and doped with BDPA radical because it gives ^31^P-NMR signal enhancements of two orders of magnitude up to a magnetic field of 14.1 T. Here we show that the large ^31^P enhancements of BDPA/PPh_3_ in benzene at 1.2 T (i) decrease when the moieties are dissolved in other organic solvents, (ii) are strongly reduced when using a nitroxide radical, and (iii) vanish with pentavalent ^31^P triphenylphosphine oxide. Those experimental observations are rationalized with numerical calculations based on density functional theory that show the tendency of BDPA and PPh_3_ to form a weak complex *via* non-covalent interaction that leads to large hyperfine couplings to ^31^P (Δ*A*_iso_ ≥ 13 MHz). This mechanism is hampered in other investigated systems. The case study of ^31^P-DNP in PPh_3_ is an important example that extends the current understanding of DNP in the liquids state: non-covalent interactions between radical and target can be particularly effective to obtain large NMR signal enhancements.

## Abstract 2

Large ^31^P-NMR enhancements are observed with DNP in PPh_3_ doped with BDPA radical, while they are reduced when a nitroxide radical or triphenylphosphine-oxide are used instead. This is due to different non-covalent radical/target molecule interactions.

## Graphical abstract

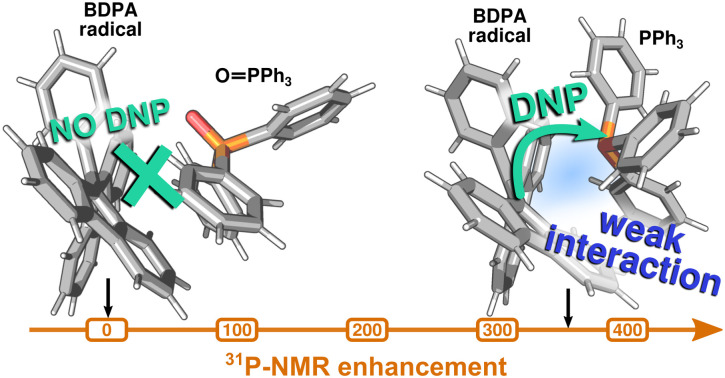

## Introduction

**Fig. 1:**
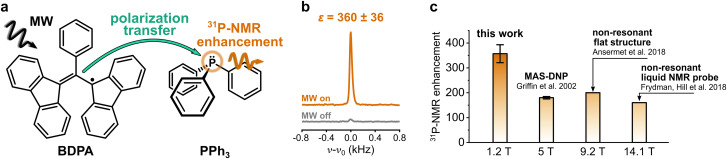
(a) Sketch of the polarization transfer process between the PA (BDPA radical) and the target molecule (PPh_3_). (b) ^31^P-NMR spectra of PPh_3_ in benzene doped with BDPA recorded at 1.2 T with and without MW irradiation. (c) ^31^P-NMR enhancements reported at different magnetic fields for PPh_3_ in benzene (fluorobenzene at 9.2 T) doped with BDPA radical.

Nuclear magnetic resonance (NMR) spectroscopy is one of the most widely used methods in analytical chemistry. It relies on the detection of small energy splittings associated with the nuclear Zeeman level and therefore suffers from low sensitivity. To overcome this limitation and expand the class of viable NMR experiments, several methods to enhance the NMR signal have been developed. Those are known as hyperpolarization methods, and they usually involve the spin polarization transfer from a highly polarized spin system to the target nuclei.^1^ Among those methods, dynamic nuclear polarization (DNP) is one of the most versatile. It uses microwave (MW) irradiation to transfer spin polarization from unpaired electron spins, usually located on an organic radical, to the nuclear spins on a target molecule (Fig. 1a).

In the liquid state, the polarization transfer is governed by a cross-relaxation process known as Overhauser effect.^2–5^ The efficiency of this process strongly depends on the choice of the polarizing agent (PA), *i.e.*, the organic radical, and of the target molecule and nucleus, as well as on the applied magnetic field. The case of ^1^H as target nucleus for DNP is rather unfavorable: ^1^H-NMR signal enhancements for water at room temperature (<45 °C) are limited to |*ε*| ≲ 30 for magnetic fields *B* ≳ 9.4 T.^6,7^ The reason for this is the dominant dipolar relaxation between the nuclear spin of ^1^H and the electron spin of the radical, which decays rapidly for increasing magnetic fields.^8^ On the contrary, ^13^C-DNP is much more effective because the dipolar relaxation is counterbalanced and overcome by scalar relaxation, a mechanism that originates from the Fermi contact interaction between the radical and the target molecule.^9^^13^C-NMR signal enhancements in liquids can be up to *ε* ∼ 1000 at a magnetic field of 3.4 T.^10^ Furthermore, recent reports showed that it is possible to obtain sizeable enhancements (>10) of ^13^C-NMR signals in a variety of small molecules at high magnetic field (9.4 T),^11^ even in water solutions.^12^ Those findings sparked new interest in the method, and now several groups are committed to tackle the open challenges to make DNP in the liquid state applicable to routine NMR spectroscopy.^13–22^ Within this context, it is important to design targets and PAs whose properties are specifically tailored to maximize the attainable NMR signal enhancements. To this aim, it is essential to identify the physical mechanisms that make the polarization transfer particularly effective.

DNP studies on target nuclei other than ^1^H and ^13^C have been only rarely reported. Here we consider the case of ^31^P, a target nucleus that is often used in NMR studies of phospholipids (*e.g.* mixtures, morphology), in metabolomics (*e.g.* ATP monitoring), and in structural investigations of DNA.^23–25^ Most studies of ^31^P-DNP date back to the early 80s, when MW and NMR technology limited the DNP measurements to magnetic fields below 1.2 T.^26–30^ The reported results show a strong dependence of ^31^P-DNP on the chemical environment of ^31^P; specifically, they observed enhancements dominated by scalar relaxation for sterically exposed trivalent phosphorus, while pentavalent phosphorus compounds show dipolar dominated enhancement.^30^ A unique case is the compound triphenylphosphine (PPh_3_) (Fig. 1a): when PPh_3_ is dissolved in benzene and doped with α,γ-Bisdiphenylene-β-phenylallyl radical (BDPA), ^31^P-NMR signal enhancements of two orders of magnitude are readily reachable, even at larger magnetic fields. ^31^P-NMR enhancements *ε* > 150 were reported for PPh_3_ at *B* ≈ 5 T,^31^ and more recently, up to *ε* ≈ 160 at 14.1 T (Fig. 1c).^13,14^ Notably, those enhancements show almost no dependence on the external magnetic field.

**Scheme 1:**
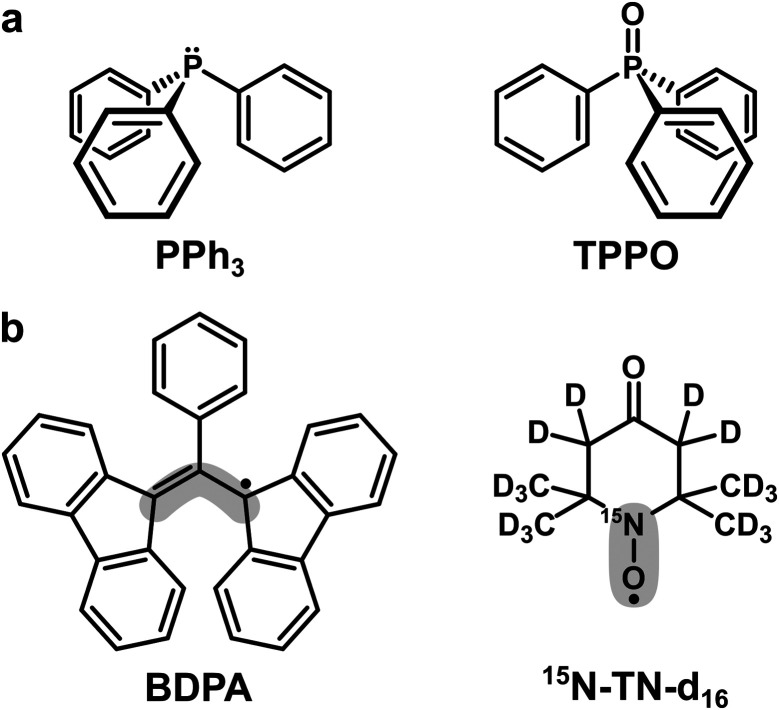
(a) Trivalent (PPh_3_) and pentavalent (TPPO) phosphorus compounds used as target molecules. (b) Organic radicals used as PAs. The grey areas mark the sites with the largest electron spin density: ∼40% for BDPA and ∼90% for ^15^N-TN-d_16_.^18^

Here we compare the performance of two polarizing agents, BDPA and the nitroxide radical TEMPONE (TN) for enhancing ^31^P-NMR signal of PPh_3_, and of its oxidized counterpart, *i.e.* triphenylphosphine oxide (TPPO) (Scheme 1). Those systems were also tested in different organic solvents, which have a significant effect on the DNP outcome. DNP measurements performed on a hybrid EPR/NMR setup operating at 1.2 T allowed us to quantify the efficiency of the Overhauser effect and show that ^31^P-DNP is particularly favourable only for the system BDPA/PPh_3_ in benzene. With numerical calculations based on density functional theory (DFT), we rationalize this observation showing that BDPA and PPh_3_ form a weak complex that favors large hyperfine couplings. Our findings corroborate the model that the formation of a complex between radical and target molecule that leads to large hyperfine coupling values is crucial to obtain large NMR signal enhancements on small molecules in the liquid state.

## Experimental methods

Triphenylphosphine (PPh_3_) and triphenylphosphine oxide (TPPO) were used as target molecules. As polarizing agents we utilized two organic radicals: BDPA (α,γ-Bisdiphenylene-β-phenylallyl, in 1 : 1 complex with benzene) and TEMPONE (TN) in its deuterated and ^15^N-labeled version (^15^N-TN-d_16_, 4-Oxo-2,2,6,6-tetramethylpiperidine-d16,1-15N-1-oxyl). Both targets and PAs were purchased from Sigma-Aldrich and used without further purification. Organic solvents chloroform (CHCl_3_), tetrachloromethane (CCl_4_), benzene, pyridine and DMSO were purchased from Merck KGaA.

The concentration of phosphorous compounds in the chosen solvent ranged between 1 M and 4 M. TPPO was soluble in CHCl_3_, pyridine and DMSO (1 M), while its solubility was too low in the less polar organic solvents benzene and CCl_4_ and prevented NMR detection at 1.2 T. The radical concentration was ∼8–10 mM and was calibrated for each sample with CW-EPR experiments. All samples were prepared in stocks of 100–550 μL, which were then degassed by freeze–pump–thaw cycles (three to five) to remove dissolved oxygen. The solutions were transferred into a glove box, where 4–4.5 μL were used to fill a quartz tube (1.6 mm outer diameter, Wilmad-LabGlass), which was then sealed with a flame. The sample preparation procedure results in an error in the radical concentration of 10% for tetrachloromethane, benzene, pyridine, and DMSO samples and 15% for chloroform samples, the latter due to a lower boiling point. Radical stability after sealing was monitored with CW-EPR. Radicals were stable in the tested solvents for the whole duration of the DNP, NMR, and EPR measurements (∼12 h).

^31^P-DNP measurements at 1.2 T were performed on a hybrid EPR/NMR system which combines a Bruker ElexSys E580 EPR spectrometer and a Bruker AVANCE III NMR console. We used a Bruker ER-5106QT/W resonator and a home built copper coil wrapped around the Q-Band quartz tube with 4 to 5 turns for NMR detection.^11^ With this arrangement, microwave and radio-frequency can be applied simultaneously, allowing for EPR, NMR, and DNP measurements on the same sample. The MW power was adjusted to avoid severe heating during MW irradiation.

Optimized geometries of the radical/target molecule pairs were computed with DFT using Orca 5.0.2^32^ with B3LYP as functional, def2-TZVPP as basis set, and the dispersion correction D3BJ. Several structures (≥12) with different starting orientations of the PA and target molecule were computed for each system; the starting orientations were chosen to take into account different approaches of the two molecules (ESI†). For each optimized geometry, we assessed the non-covalent interactions by calculating the interaction energy *E*_int_, defined as the difference between the electronic energy of the dimer and the electronic energy of the two monomers.^33^ The values were corrected for the basis set superposition error with the Boys-Bernardi procedure (ESI†).^34–37^ The calculations were performed in vacuum as well as with the implicit solvation model C-PCM^38,39^ for benzene and chloroform, using the same starting geometries. We calculated the isotropic hyperfine coupling to ^31^P using EPR-III basis set^40^ for H, C, N, and O atoms and IGLO-II^41^ for P atom.

## Results and discussion

**Fig. 2:**
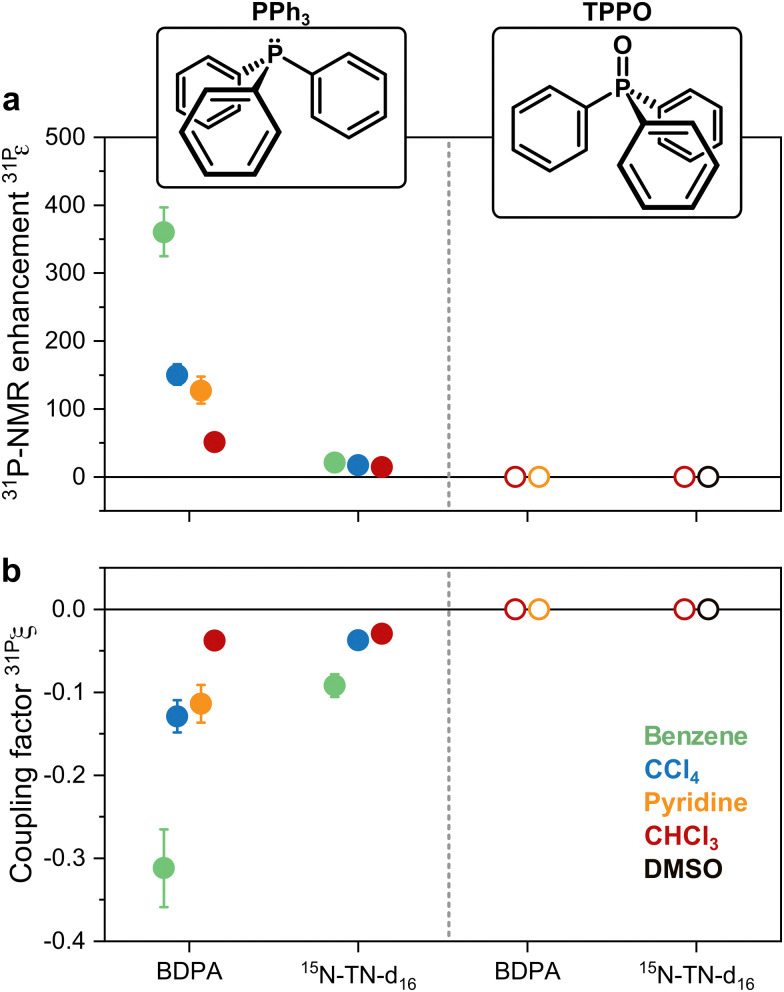
(a) ^31^P-NMR enhancements recorded at 1.2 T and (b) coupling factors calculated with eqn (1) for both PPh_3_ and TPPO doped with BPDA and ^15^N-TN-d_16_ in different solvents.

^31^P-NMR signals of PPh_3_ and TPPO in various solvents were recorded with (DNP) and without MW irradiation (thermal) at a magnetic field of 1.2 T. ^31^P-NMR signals were integrated to calculate the enhancements *ε* = *I*_DNP_·*n*_thermal_/(*I*_thermal_·*n*_DNP_), where *I* is the integral and *n* is the number of scans. Fig. 2a reports the enhancements of the investigated systems. In PPh_3_, the highest ^31^P-NMR signal enhancement, *i.e.*^31P^*ε* = 360 ± 36, has been obtained in benzene and when using BDPA. The radical ^15^N-TN-d_16_ is not as efficient, and gives ^31^P-NMR signal enhancements that are a factor of 5 to 10 smaller, with a maximum of ^31P^*ε* = 21 ± 3 for PPh_3_ in benzene. No ^31^P-NMR signal enhancements were observed on the TPPO molecule with all utilized solvents and PAs. Earlier reports^30^ suggest that the large enhancements observed in PPh_3_ could be due to the lone pair of the trivalent ^31^P, which tends to coordinate with the unpaired electron of the radical. On the contrary, ^31^P in TPPO is in the pentavalent configuration, which hampers this mechanism.

The enhancements give only partial insight into the efficiency of ^31^P-DNP. Indeed, ^31P^*ε* is determined by several parameters, as given by the Overhauser equation:^2,4^1
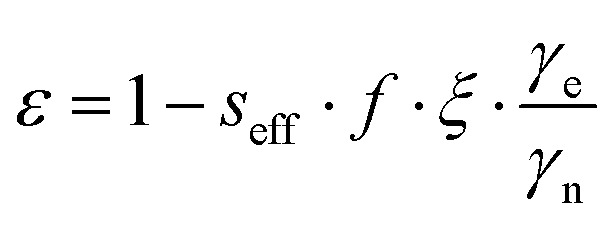
where *ε* is directly proportional to: (i) the effective saturation factor *s*_eff_, quantifying how much of the EPR line is saturated; (ii) the leakage factor *f*, which stands for the amount of paramagnetic relaxation over the total nuclear relaxation; (iii) the ratio of electron (*γ*_e_) and nuclear (*γ*_n_) gyromagnetic ratios, which is *γ*_e_/*γ*_n_ ∼ 1626 for ^31^P; (iv) the coupling factor *ξ* that accounts for cross-relaxation transition rates between the electron and the nuclei and ultimately gives the efficiency of the polarization transfer.^5,42,43^ Therefore, to compare the DNP efficiency among the tested systems, it is important to quantitatively access the coupling factor *ξ* with eqn (1) once *f* and *s* are known.

The leakage factor *f* depends on the nuclear relaxation times of the target nucleus measured with (*T*_1,n_) and without radical (*T*_1,dia_), and *f* = 1 − *T*_1,n_/*T*_1,dia_. *f* depends ultimately on the concentration of the PA (*c*_PA_) and for our samples is *f* ∼ 0.8 or larger for *c*_PA_ ∼ 8–10 mM (Table 1). The effective saturation factor *s*_eff_ depends on the type of radical chosen as PA and can vary significantly. The EPR spectrum of BDPA shows a hyperfine structure that arises from the coupling to the protons on the diphenylene and phenyl rings (ESI† Fig. S2). At 34 GHz and *c*_PA_ ∼ 10 mM the lines are merged into one and the total spectral width is ∼1 mT; this ensures an almost complete saturation of the EPR line when MW irradiation is applied on resonance with the center of the line. On the contrary, the spectrum of ^15^N-TN-d_16_ consists of two sharp lines separated by ∼2 mT stemming from the hyperfine coupling with the ^15^N nucleus. When one of the two lines is irradiated on resonance (as in a DNP experiment), the other line is only partially saturated *via* a mechanism known as ELDOR effect.^44–46^ The saturation factors were measured with an ELDOR (electron double resonance) experiment (ESI†) and, under similar experimental conditions, *s*_eff_ is *s*_eff_ > 0.8 for BDPA, while it is limited to *s*_eff_ < 0.35 for ^15^N-TN-d_16_ (Table 1). The leakage and the saturation factors together cannot explain the difference in enhancements among the tested systems.

**Table 1:** Overhauser parameters of ^31^P-DNP of triphenylphosphine (PPh_3_) for the two different PAs in different organic solvents at 1.2 T. The leakage factor *f* was calculated as *f* = 1 − *T*_1,n_/*T*_1,dia_ using *T*_1,dia_ = 10.9 s for PPh_3_ in CHCl_3_, 19.0 s in CCl_4_, 20.5 s in benzene and 21.6 s in pyridine, respectively. Errors are 10% for *c*_PA_ (15% for CHCl_3_ samples), 10% for *c*_PPh3_ (15% for CCl_4_), 10% for *T*_1,n_, *T*_Buildup_, *f*, *s*_eff_, and *ε*, and is 15% for *ξ* (15% for *ε* and 20% for *ξ* in pyridine). Errors are increased for *ε* and *ξ* to 15% and 25%, respectively if *T*_1,n_ ≠ *T*_Buildup_, which indicates sample heating during MW irradiation

PA	Solvent	*c*_PA_ (mM)	*c*_PPh3_ (M)	*T*_1,n_ (s)	*T*_Buildup_ (s)	*f*	*s*_eff_	*ε*	*ξ*
BDPA	CHCl_3_	10	4	1.2	1.1	0.89	0.91	51	−0.038
BDPA	Pyridine	10	2	3.7	3.8	0.83	0.82	127	−0.114
BDPA	CCl_4_	10	2	3.8	4.5	0.80	0.90	150	−0.129
BDPA	Benzene	10	2	6.1	8.4	0.70	1.00	360	−0.312
^15^N-TN-d_16_	CHCl_3_	10	4	2.3	1.3	0.79	0.34	14	−0.030
^15^N-TN-d_16_	CCl_4_	10	2	2.3	2.0	0.88	0.30	17	−0.037
^15^N-TN-d_16_	Benzene	8	2	3.5	3.4	0.83	0.16^a^	21	−0.092

a For this sample, the MW power was reduced to avoid excessive sample heating.

With those values of *f* and *s*_eff_ and eqn (1), one can calculate the coupling factor ^31P^*ξ* (Fig. 2b). The quantification of this experimental parameter leads to three important observations: (1) BDPA is a better PA than ^15^N-TN-d_16_ for ^31^P and is characterized by a more efficient polarization transfer, *i.e.* |^31P^*ξ*_BDPA_| > |^31P^*ξ*_TN_|: this is rather surprising because nitroxide radicals are superior PAs both for ^1^H and ^13^C DNP;^18^ (2) no enhancements are observed on ^31^P-TPPO at 1.2 T and ^31P^*ξ* ∼ 0 independently of the solvent; (3) the solvent influences the ^31^P-DNP performance: in particular, benzene is the best solvent, while CCl_4_, pyridine, and chloroform follow in this order, for both BDPA and TN.

In the following, we rationalize those observations and investigate the interactions between target molecule and PA that could affect the polarization transfer process. From previous studies^5,43^ it is known that, when the enhancements are positive (^31P^*ε* > 0, and therefore ^31P^*ξ* < 0), the scalar (or Fermi contact) interaction between the electron and the nucleus is dominant over the dipolar one. The scalar interaction drives electron-nuclear cross-relaxation through modulation of the isotropic hyperfine coupling (*A*_iso_) between the electron spin of the radical and the nuclear spin of the target. In the case of small molecules interacting with organic radicals in liquids at room temperature, those modulations arise from a collisional process.^47^ Particularly, one has *A*_iso_ ≠ 0 during the collision and *A*_iso_ = 0 when the two molecules diffuse apart. The collisional process is described by the following spectral density:^47,48*a*^2
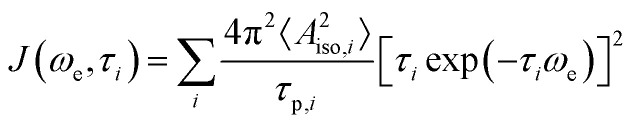
where the index *i* stands for the *i*-type of collision. The duration of each collision is *τ*, the collision rate is 1/*τ*_p_, and the hyperfine coupling is *A*_iso_ (in Hz in eqn (2)). This analytical model is a good approximation of more sophisticated numerical simulations^48*a,b*^ and can be effectively used to interpret DNP data. Furthermore, as earlier proposed by the group of Dorn,^9*c,d*^ the hyperfine coupling calculated with DFT correlates with the scalar enhancement observed experimentally in a variety of compounds, provided that the timescale of the collisional process (*τ*_*i*_, *τ*_p,*i*_) is comparable. Here we utilize a similar methodology to examine the interaction between target molecules (PPh_3_ and TPPO) and the radicals (BDPA and TN).

**Fig. 3:**
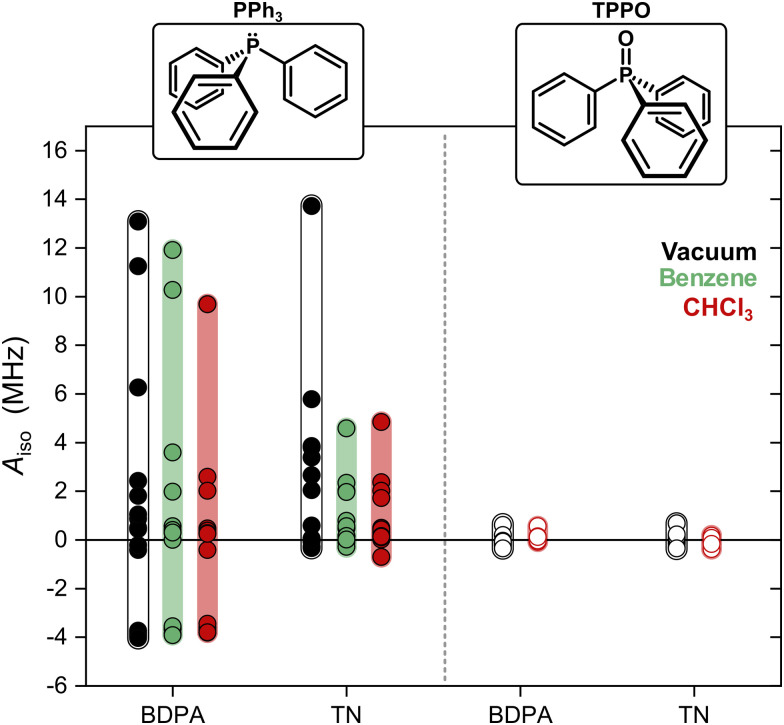
Hyperfine coupling *A*_iso_ calculated for each optimized structure of the investigated complexes. 15 structures were computed for both PPh_3_ systems, and 12 for TPPO. The circles are calculated values and they partially overlap. Color bands are visual aids.

We computed optimized geometries (up to 15 optimized structures for each complex for each solvent) and isotropic hyperfine couplings of the complexes BDPA/PPh_3_, TN/PPh_3_, BDPA/TPPO, and TN/TPPO. The results are summarized in Fig. 3, where each point represents *A*_iso_ for each optimized structure in vacuum, benzene, and chloroform. When PPh_3_ interacts with the BDPA radical, *A*_iso_ spans the largest range, Δ*A*_iso_ ∼ 17 MHz, going from negative (*A*_iso_ ∼ −4 MHz) to positive (*A*_iso_ > 10 MHz) in vacuum. The range is smaller for the system TN/PPh_3_, Δ*A*_iso_ ∼ 14 MHz, and it is further reduced to Δ*A*_iso_ ∼ 5 MHz when the solvent model is used. For TPPO, the hyperfine couplings are more than 10 times smaller and span the range −0.4 MHz < *A*_iso_ < 0.7 MHz.

**Fig. 4:**
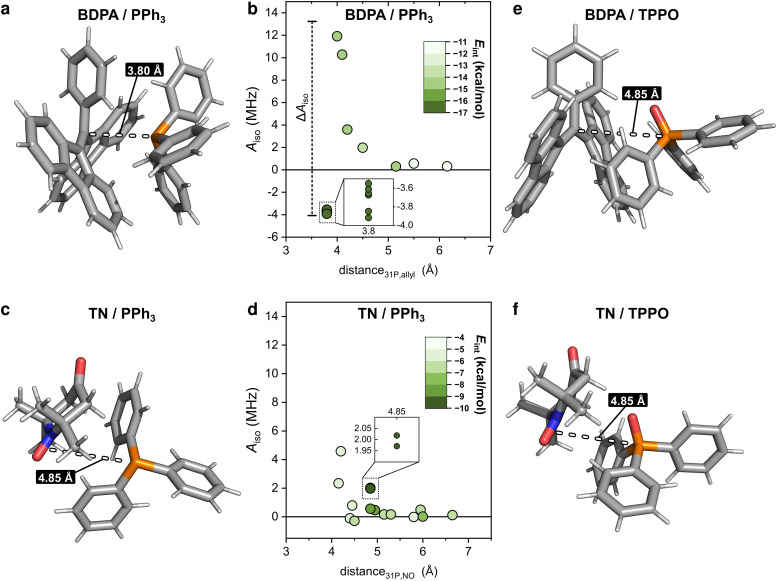
(a) Structure of the complex BDPA/PPh_3_ in benzene corresponding to the minimum interaction energy *E*_int_ = −16.3 kcal mol^−1^ and with *A*_iso_ = −3.7 MHz where the lone pair of the ^31^P atom is pointing to the allyl group of the BDPA. (b) Hyperfine coupling *A*_iso_ calculated for each of the optimized structures in benzene and plotted as a function of the distance between ^31^P and the allyl group of the BDPA. The distance is the mean of the distances between ^31^P and the two closest carbons of the ally group of BPDA. The color map shows the interaction energy *E*_int_ of the complex. (c) Structure of the complex TN/PPh_3_ in benzene with minimum interaction energy *E*_int_ = −9.5 kcal mol^−1^ and with *A*_iso_ = 2.02 MHz. (d) Hyperfine coupling *A*_iso_ calculated for each optimized structure in benzene and plotted as a function of the distance between ^31^P and the NO group of TN. (e) Structure of the complex BDPA/TPPO in CHCl_3_ with minimum interaction energy *E*_int_ = −15.1 kcal mol^−1^ and with *A*_iso_ = −0.07 MHz. (f) Structure of the complex TN/TPPO in chloroform with minimum interaction energy *E*_int_ = −9.4 kcal mol^−1^ and with *A*_iso_ = −0.02 MHz. Atom colour code: H white, C grey, N blue, O red, P orange.

To clarify why the two radicals lead to a different *A*_iso_ to the ^31^P of PPh_3_, we took a closer look at the calculated geometries. By considering the pairs BDPA/PPh_3_, one notices that large hyperfine couplings (|*A*_iso_| > 3 MHz) are observed when the lone pair of ^31^P is pointing directly to the allyl group of BDPA, which carries the majority of the electron spin density (∼40%) (Fig. 4a and Scheme 1).^18^ This configuration is unusual because the accessibility of the allyl group by small solvent molecule is limited.^18^ The interaction energy *E*_int_ calculated for each BDPA/PPh_3_ optimized structure show that complexes with short ^31^P-allyl group distances (*d* ≤ 4 Å) and large hyperfine couplings (|*A*_iso_| > 3 MHz) are energetically favored over other geometries where ^31^P is further away from the allyl group (Fig. 4b and Fig. S4, ESI†). The same is found for calculations performed in vacuum, benzene, and chloroform.

We performed a similar analysis for the system TN/PPh_3_. In terms of distance, ^31^P cannot get as close to the electron spin density localized on the NO group of TN (*d* > 4.2 Å in benzene) (Fig. 4c). The interaction energy *E*_int_ has a minimum at distances *d* ∼ 4.85 Å, which corresponds to a hyperfine coupling |*A*_iso_| ∼ 2 MHz (Fig. 4d and Fig. S4, ESI†). Therefore, TN/PPh_3_ show a non-covalent interaction that is less efficient in terms of hyperfine coupling. Additional calculations of *E*_int_ were performed with fixed values for the distance between ^31^P and the electron spin density (both for BDPA and TN), and confirm the tendency of PPh_3_ to approach BDPA, and specifically near the allyl group, while this is not the case for the pair TN/PPh_3_ (Fig. S6a, ESI†). Those results indicate that the large ^31^P-NMR enhancements observed on PPh_3_ doped with BDPA are facilitated by a non-covalent interaction that leads to large values of hyperfine coupling *A*_iso_, which in turn renders the scalar relaxation from the collisional process more efficient (eqn (2)).

In regard to TPPO, the absence of enhancements must be a consequence of particularly small hyperfine couplings (Fig. 3). The oxygen atom bound to ^31^P hampers a close approach between the ^31^P of TPPO and BDPA (Fig. 4e) and leads to low hyperfine coupling values (|*A*_iso_| < 0.7 MHz). In the case of TN, TPPO reaches a distance ^31^P-NO group *d* ≥ 4.2 Å in both vacuum and chloroform, which is similar to the distances obtained for PPh_3_ (Fig. 4f). Nevertheless, the hyperfine coupling |*A*_iso_| remains below 0.7 MHz. As already observed for ^13^C-DNP of carbonyl groups,^9*c,d*,10^ the oxygen atom might be responsible for withdrawing electron spin density from ^31^P,^49^ which results in lower *A*_iso_ values thus decreasing the attainable enhancements.

The last remark concerns the role of the solvent. Recent experimental and theoretical studies on DNP mechanisms have been focused on the interaction of only two moieties, i.e the radical and a small molecule that is at the same time the solvent and the target molecule.^7,9–11,48^ Our experimental data show that a third player, *i.e.* the solvent, has a considerable effect in ^31^P-DNP and possibly contributes to the spin polarization transfer. In the context of DNP, the solvent (i) determines the degree of MW absorption, and (ii) when diffusivity increases, dipolar and scalar relaxations increase.^50^ The diffusion coefficient for benzene at 298 K is *D* = 2.2 × 10^5^ cm^2^ s^−1^,^51^ while it is lower for CHCl_3_ (*D* = 2.14 × 10^5^ cm^2^ s^−1^),^17^ CCl_4_ (*D* = 1.4 × 10^5^ cm^2^ s^−1^),^51^ and pyridine (*D* = 1.54 × 10^5^ cm^2^ s^−1^).^52^ Although a faster collision rate enabled by a larger *D* might favor ^31^P-DNP in benzene, the minor differences in *D* cannot explain the large differences observed in coupling factors. The solvent might affect the kinetics of the interaction between radical and target molecule, either stabilizing a non-covalent interaction or preventing its formation. Despite the presented numerical analysis considers the effect of the solvent through an implicit solvation model, it is not sufficient to gain insight in these mechanisms. To the best of our knowledge, more sophisticated simulations tools based on molecular dynamics and used for other systems^48^ are not yet available for large radicals like BDPA. Such investigations will be the subject of future works.

## Conclusions

We investigated large ^31^P-NMR enhancements that have been observed from 1.2 T (this study) up to 14.1 T^13,14,31^ on the system BDPA/PPh_3_. Our DNP data recorded on a hybrid EPR/NMR instrument operating at 1.2 T show that those enhancements are attenuated when TEMPONE radical is used as a PA, and vanish when triphenylphosphine oxide (TPPO) is the target molecule. The experimental observations were interpreted in the context of the collisional model for scalar relaxation with the support of DFT calculations and show that PPh_3_ forms a weak complex with the polarizing agent BDPA, which favors large hyperfine couplings. This mechanism is precluded when ^31^P is in the pentavalent configuration or when a nitroxide is used. This finding provides a novel aspect within the context of DNP in the liquid state and shows how the choice of the optimal PA is strongly dependent on the chemical environment of the target nucleus. We foresee that the design of future PAs for liquid DNP, possibly supported by DFT calculations, will use non-covalent interactions between target molecule and PA as an effective strategy to boost the NMR enhancements.

## Data availability

Original data associated with Fig. 2 (ELDOR spectra, NMR spectra, NMR relaxation data), and output files of all geometries optimizations shown in Fig. 3 and 4 can be downloaded free of charge from the Göttingen Reseach Online website (DOI: 10.25625/A5WDZW).

## Conflicts of interest

There are no conflicts to declare.

## Supplementary Material

CP-025-D2CP04092A-s001
