# Supplementary material for: Large 31P-NMR enhancements in liquid state dynamic nuclear polarization through radical/target molecule non-covalent interaction
Source: Phys Chem Chem Phys. 2022 Dec 13;25(1):822–8. doi: 10.1039/d2cp04092a (PMC9768845; doi:10.1039/d2cp04092a)
Supplement: CP-025-D2CP04092A-s001 [file CP-025-D2CP04092A-s001.pdf]

This journal is © the Owner Societies 2022

# - Supplementary Information -

Large ^{31}P-NMR enhancements in liquid state dynamic nuclear polarization through radical/target molecule non-covalent interaction

Maik Reinhard,[a,b] Marcel Levien,[a,b] Marina Bennati,[a,b] and Tomas Orlando[a]

[a] ESR Spectroscopy Group, Max Planck Institute for Multidisciplinary Sciences, Am Faßberg 11, Göttingen, Germany
[b] Department of Chemistry, Georg-August-University, Tammannstraße 4, Göttingen, Germany

Contents

S1. Overhauser experiments and parameters 1
S2. Quantum chemistry calculations 4

S1. Overhauser experiments and parameters

^{31}P-DNP measurements at 1.2 T were performed on a Bruker ElexSys E580 EPR spectrometer combined with an AVANCE III ^{1}H 300 MHz NMR console. A Bruker ER-5106QT/W cw resonator was employed. A home built copper coil was wrapped around the Q-Band quartz tube with 4 to 5 turns for NMR detection. This is reducing the Q-value of the resonator but still allows for tuning and matching of the cavity. The MW power was tuned to prevent severe heating of the sample during MW irradiation.

Nuclear T_1

For the measurements of $T_{1,\mathrm{dia}}$ (i.e. longitudinal nuclear relaxation time of ^{31}P of phosphorus compounds without radical) larger sample volumes were necessary to detect the NMR signals without DNP. Therefore, we used a setup that can accommodate a larger sample volume, that is 100 µL inside a 3 mm glass tube instead of ~ 4 µL used during the DNP experiments at 1.2 T. The phosphorus compounds were dissolved in the different organic solvents with the respective concentration as in the DNP samples. The solutions were degassed by freeze-pump-thaw cycles (four to six) and afterwards the tube was sealed with a flame.

We utilized an EPR resonator with Electron Nuclear Double Resonance (ENDOR) capabilities for NMR detection (Bruker EN4118X-MD-4) tuned at 13.7 MHz, which corresponds to a magnetic field of 0.8 T. $T_{1,\mathrm{dia}}$ of the phosphorus compounds in different solvents were measured with a saturation recovery experiment (with 2 saturation pulses each of duration of $(\pi/2)_{\mathrm{sat}}$= 6.2 – 6.7 µs, $\pi/2 = 6 - 6.5\ \mu\mathrm{s}$, $P_{\mathrm{RF}} = 60\ \mathrm{W}$). $T_{1,\mathrm{dia}}$ was obtained by fitting the NMR signals (area) recorded as a function of recovery time with the exponential function $A - B \cdot \exp(-t/T_{1,\mathrm{dia}}))$, where A, B, and $T_{1,\mathrm{dia}}$ are fitting parameters. A recovery curve for ^{31}P of PPh_3 in benzene is shown in Figure S1e with the pulse sequence.

Enhancement ε

^{31}P-NMR signal enhancements were obtained by the ratio of the area of the NMR signal with and without MW irradiation. Enhanced NMR spectra were obtained with 2 – 8 scans, whereas for Boltzmann spectra 150 – 900 scans were needed to obtain a moderate signal-to-noise ratio. The recycle delay of the signal averaging was ~ $5 \cdot T_{1,\mathrm{n}}$. The signal enhancement ε was calculated with:

$$\varepsilon = \frac{I_{\mathrm{DNP}}}{I_{\mathrm{thermal}}} \cdot \frac{n_{\mathrm{thermal}}}{n_{\mathrm{DNP}}}, \qquad \text{(S1)}$$

where I_{DNP} and I_{thermal} are the areas of the NMR signal with and without MW irradiation and n_{DNP} and n_{thermal} are the number of scans with and without MW irradiation. DNP enhanced and Boltzmann NMR spectra and pulse sequence are displayed in Figure S1a for ^{31}P of PPh_3 in benzene doped with BDPA.

Saturation factor s_{eff}

Effective saturation factors were obtained by performing an ELDOR (Electron Nuclear Double Resonance) experiment. There, a long saturation pulse (5 µs) is swept through the EPR spectrum, while the EPR signal is detected on one of the EPR transitions. If the ELDOR pulse is on resonance with either one of the EPR lines, a drop in signal intensity is observed. The saturation factors s_i can be obtained from these signal drops. The effective saturation factor s_{eff} can then be calculated for a n-line system with the following equation [1]:

$$s_{\mathrm{eff}} = \frac{1}{n} \cdot \sum_{i=1}^{n} s_i \, . \qquad \text{(S2)}$$

In Figure S1b, an ELDOR spectrum is shown for BDPA in benzene with PPh_3 and the ELDOR pulse sequence in the inset.

Leakage factor f

The leakage factor f was calculated as:

$$f = 1 - \frac{T_{1,\mathrm{n}}}{T_{1,\mathrm{dia}}}, \qquad \text{(S3)}$$

where $T_{1,\mathrm{n}}$ and $T_{1,\mathrm{dia}}$ are the nuclear relaxation times with and without the presence of the paramagnetic species. Due to the low sensitivity, $T_{1,\mathrm{n}}$ was measured with a polarization decay experiment, where a short pre-polarization MW pulse (2 – 6 s) was applied before NMR detection. The delay time between pre-pulse and detection was incremented and the decay of the NMR signal (area) was fitted with the exponential function $A \cdot \exp(-t/T_{1,\mathrm{n}})$ where A and $T_{1,\mathrm{n}}$ are fitting parameters. The build-up times T_{Buildup} were measured by increasing the MW irradiation time and T_{Buildup} were again obtained by fitting the NMR signals (area) with the exponential function $A - B \cdot (1 - \exp(-t/T_{\mathrm{Buildup}}))$, where A, B, and T_{Buildup} are fitting parameters. T_{Buildup} and $T_{1,\mathrm{n}}$ curves for ^{31}P of PPh_3 in benzene doped with BDPA are shown in Figure S1c,d with their respective pulse sequences.

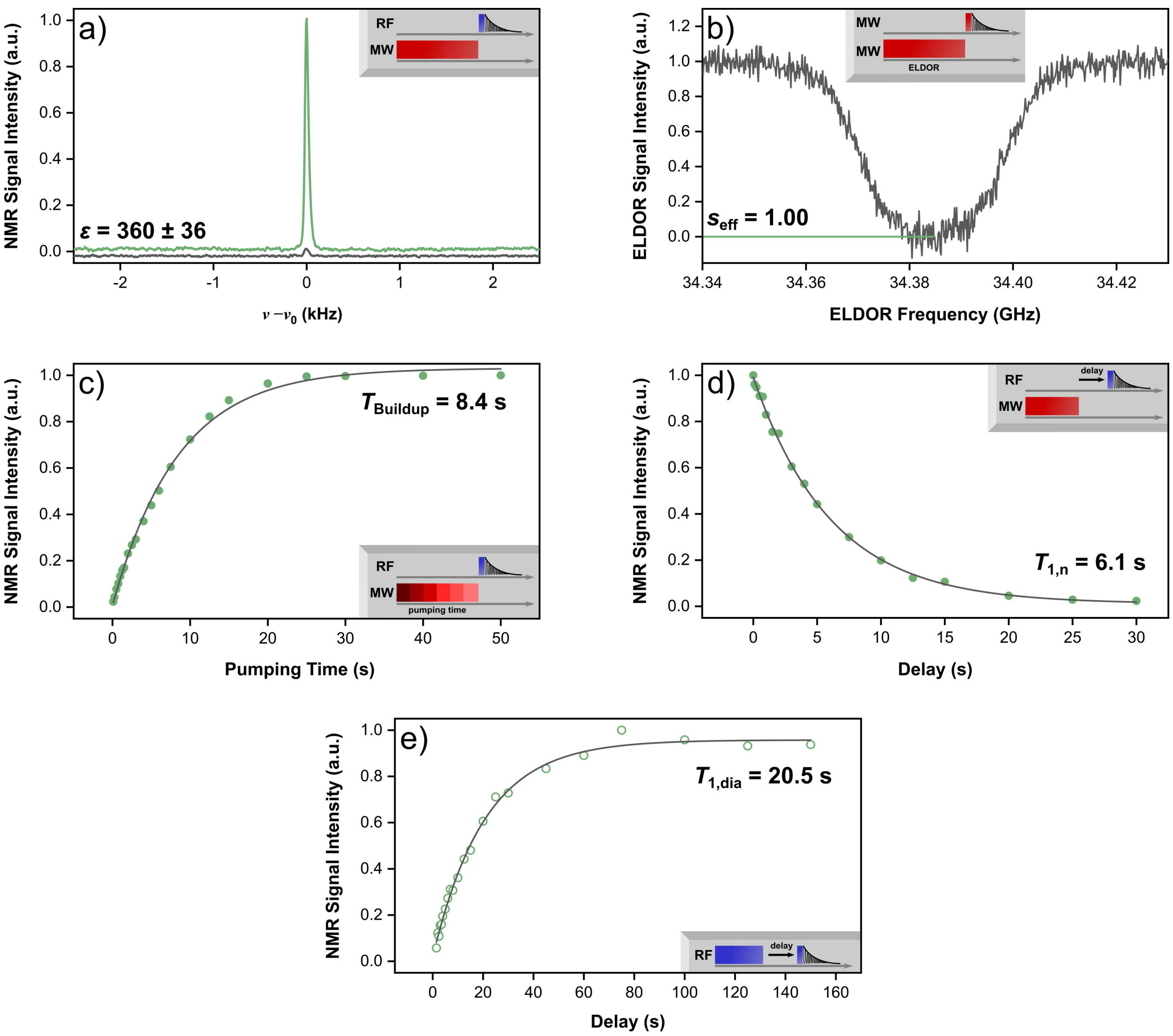

Figure S1. Experimental data of triphenylphosphine (PPh_3) in benzene doped with BDPA as PA at 1.2 T. Respective pulse sequences are shown in the insets. **a)** DNP enhanced ^{31}P-NMR signal (green) and Boltzmann NMR spectrum without MW irradiation (grey). **b)** Saturation curve (grey) of BDPA in benzene with PPh_3 and s_{eff} marked at signal intensity drop. **c)** Build-up measurement with increasing MW pumping time. **d)** Polarization decay experiment to obtain nuclear relaxation time $T_{1,n}$ with presence of PA. **e)** Determination of $T_{1,dia}$ via saturation recovery experiment of PPh_3 in benzene without PA at 0.8 T (different sample than a – d indicated by green rings as data points). Grey curves in c – e are the mono exponential fits to obtain $T_{Buildup}$, $T_{1,n}$ and $T_{1,dia}$.

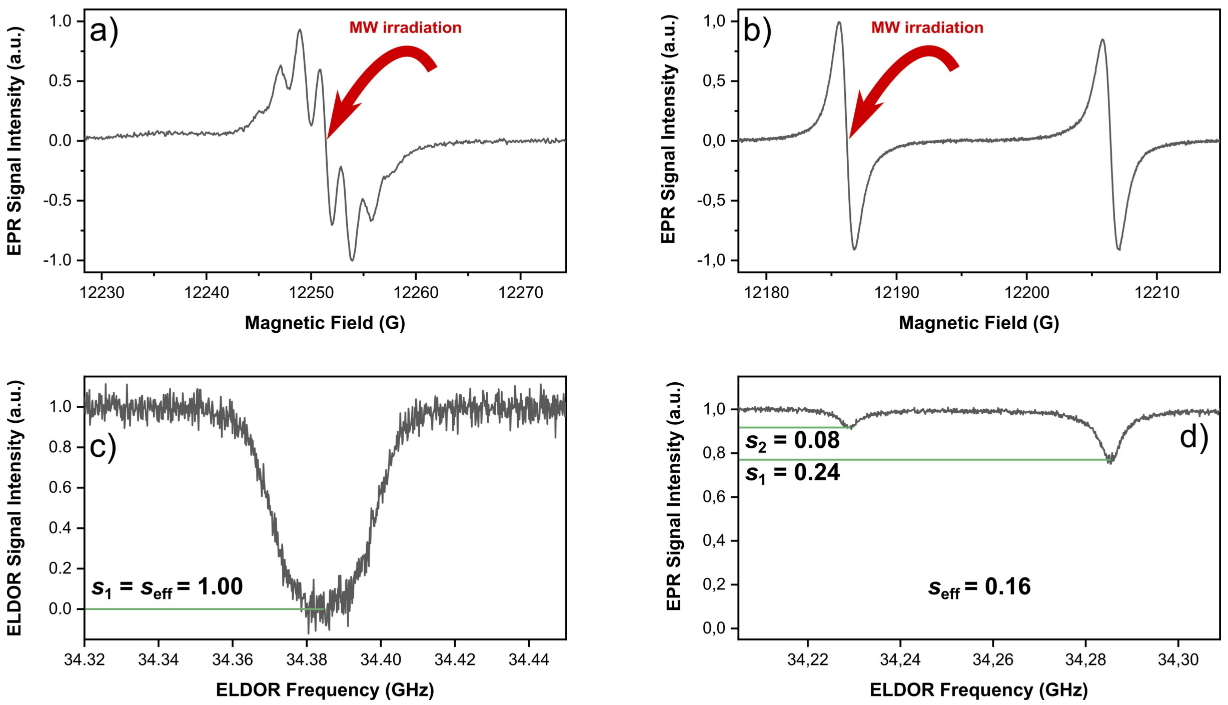

Figure S2. a, b) CW-EPR spectra at 1.2 T of BDPA and ^{15}N-TN-d_{16} in benzene (10 mM and 8 mM), respectively, with PPh_3 (2 M) at room temperature. Red arrows indicate detection field value for ELDOR experiment. **c, d)** Saturation curves (grey) of BDPA and ^{15}N-TN-d_{16} of the same sample as the CW measurements and s_i marked at the respective signal intensity drops. Note that in the ELDOR experiment of ^{15}N-TN-d_{16} the MW power was reduced to avoid excessive sample heating.

S2. Quantum chemistry calculations

Geometry optimization

Geometry optimizations of the polarizing agent structures, target molecule structures, and complexes have been computed with Orca 5.0.2 [2]. The calculations were performed at B3LYP level of theory and the def2-TZVPP basis set was used. Resolution-of-the-identity, chains-of-spheres approximations (RIJCOSX with def2/J auxiliary basis set) and the dispersion correction (D3BJ) were also employed. The optimization procedure (TIGHTOPT) was used and for the SCF very tight convergence criteria (VERYTIGHTSCF). For each complex, different orientations of the PA with respect to the target molecule were chosen as starting point for the geometry optimization (Figure S3). The optimizations were computed in vacuum, benzene, and chloroform using the same starting structures [3]. Afterwards, the isotropic hyperfine coupling to ^{31}P was calculated for each optimized geometry using EPR-III basis set [4] for H, C, N, and O atoms and IGLO-II [5] for P atom.

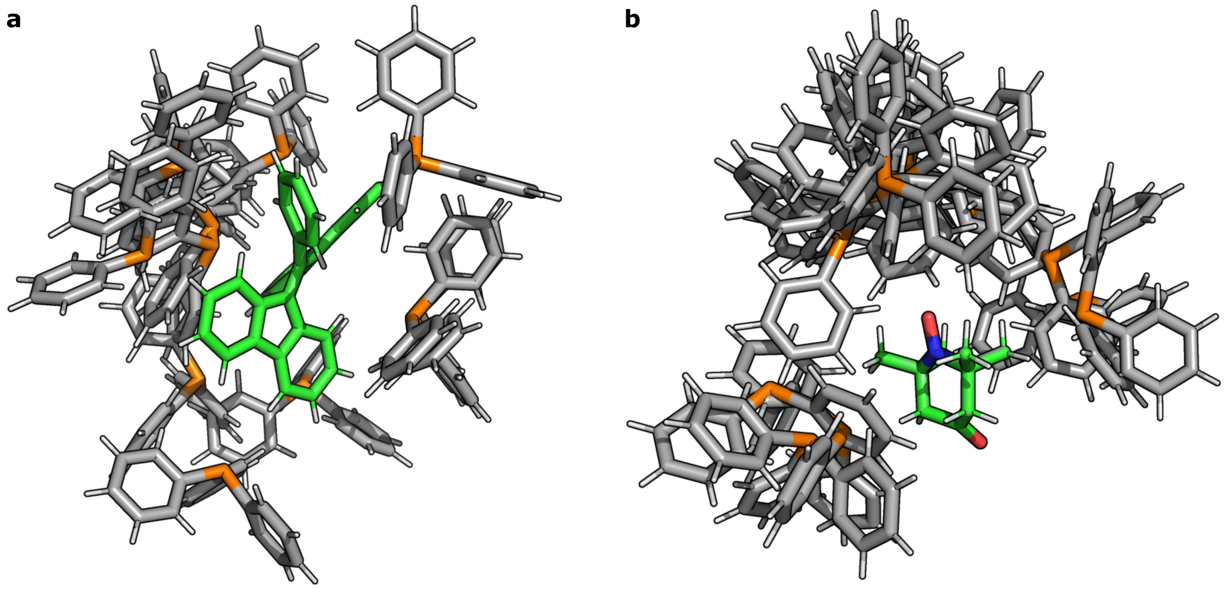

Figure S3. Starting structures for the complexes **a)** BDPA/PPh_3 (12 geometries of the 15 calculated) and **b)** TN/PPh_3 (15 geometries). The radical structures are aligned and superimposed. Color code: H white, C-target grey, C-radical green, N blue, O red, P orange.

Interaction energy

Interaction energies were computed as described by Boys and Bernardi in Ref. [6]. Interaction energies for each complex are:

$$E_{\mathrm{int}} = E_{RT}^{RT}(RT) - E_{T}^{T}(T) - E_{R}^{R}(R) - \left[E_{R}^{RT}(RT) - E_{R}^{RT}(R) + E_{T}^{RT}(RT) - E_{T}^{RT}(T)\right]$$

where the index R stands for 'radical', and T stands for 'target molecule'. The terms are:

- $E_{RT}^{RT}(RT)$: single point energy obtained optimizing the complex radical / target molecule.
- $E_{R}^{R}(R)$ and $E_{T}^{T}(T)$: single point energy of the optimized geometries of the radical and of the target molecule, respectively.
- $E_{R}^{RT}(R)$ and $E_{T}^{RT}(T)$: single point energies of the radical and the target molecule, respectively, with the geometries that they have in the complex.
- $E_{R}^{RT}(RT)$ and $E_{T}^{RT}(RT)$: single point energies of the radical and the target molecule, respectively, with the geometries that they have in the complex but computed with the full basis set of the complex.

Further details are reported in Section 8.1.6 of the manual or the ORCA software, version 5.0.3.

Figure S4 shows the hyperfine coupling A_{iso} calculated for each optimized structure in vacuum, benzene, and chloroform and plotted as a function of the distance between ^{31}P and the electron spin density on the radical. The interaction energy E_{int} is shown as color map. Figure S5 shows the interaction energy of each of the optimized complex radical/PPh_3 as a function of the distance between ^{31}P and the site where the electron spin density is localized.

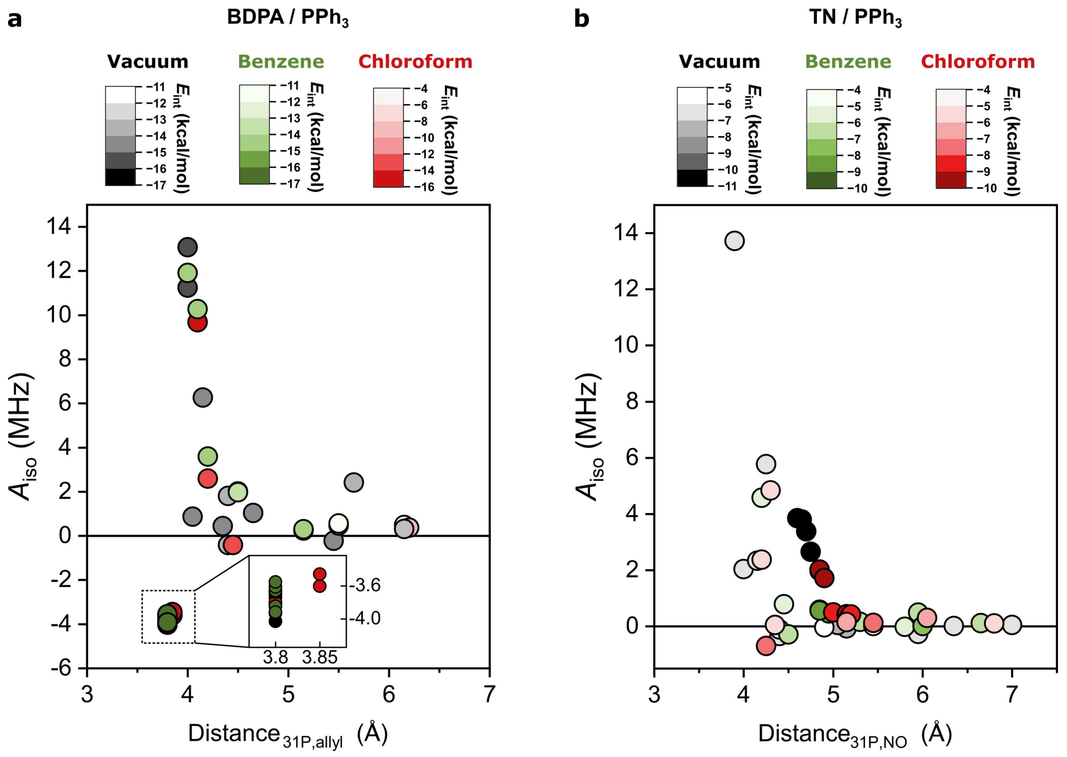

Figure S4. **a)** Hyperfine coupling A_{iso} calculated for each of the optimized structures vacuum, benzene, and chloroform plotted as a function of the distance between ^{31}P and the allyl group of the BDPA. The distance is the mean of the distances between ^{31}P and the two closest carbons of the ally group of BPDA. **b)** Same calculations for the complex TN/PPh_3; In this case, we considered the mean distance between ^{31}P and the NO group of TN radical.

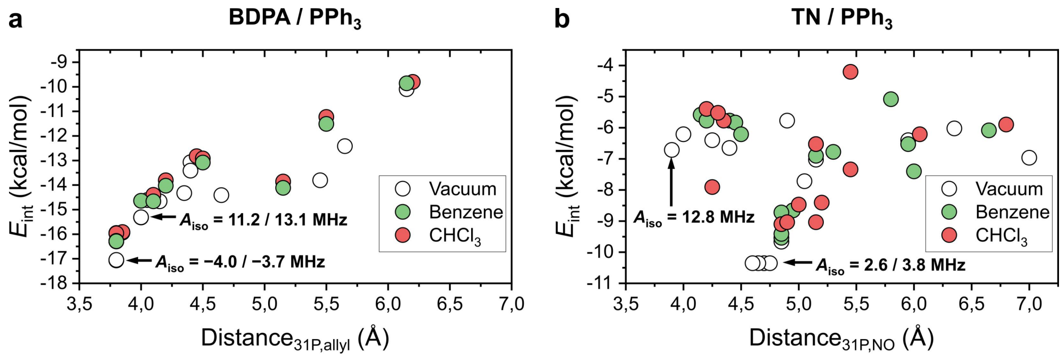

Figure S5. **a)** Interaction energy of the optimized complexes BDPA/PPh_3 as a function of the mean distance between ^{31}P and the two closest atoms of the allyl group of BDPA. **b)** Interaction energy of TN/PPh_3 as a function of the mean distance between ^{31}P and the NO group.

Additional series of geometry optimization calculations were run with fixed distances between ^{31}P and the radical site with the largest spin density (Figure S6). For BPDA, we fixed the distance between ^{31}P and the C atom at the center of the allyl group, while for TN we consider the distance between ^{31}P and the oxygen atom of the NO group. Figure S6 shows the interaction energies (in vacuum) of the compounds and the tendency of BDPA/PPh_3 to for a complex.

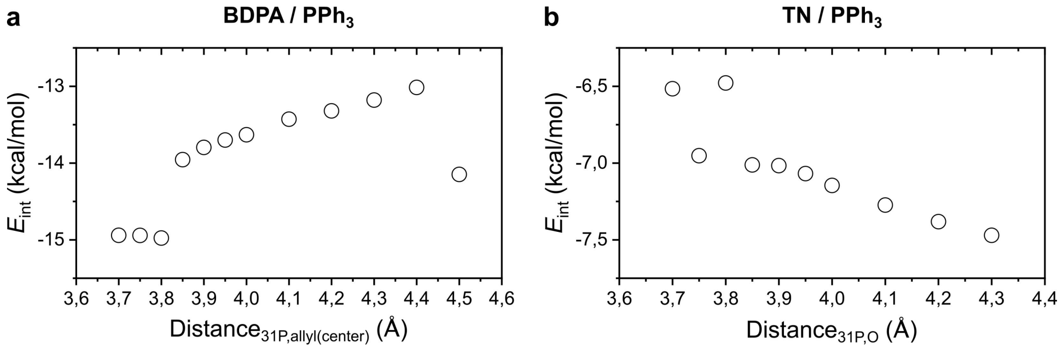

Figure S6. **a)** Interaction energy calculated for the optimized geometry of BDPA/PPh_3 in vacuum when the distance ^{31}P - C center of the allyl group (on the x-axis) is fixed. **b)** Energy calculated for the optimized geometry of TN/PPh_3 when the distance ^{31}P - O (on the x-axis) is fixed.

References

1) M. T. Türke, M. Bennati, *Phys. Chem. Chem. Phys.*, 2011, **13**, 3630.

2) F. Neese, *Wiley Interdiscip. Rev.: Comput. Mol. Sci.*, 2012, **2**, 73–78.

3) V. Barone, M. Cossi, *J. Phys. Chem. A* 1998, **102**, 11, 1995–2001.

4) V. Barone, in *Recent Advances in Density Functional Methods, Part I,* Ed. D. P. Chong (World Scientific Publ. Co., Singapore, 1996).

5) W. Kutzelnigg, U. Fleischer and C. van Wüllen, Shielding Calculations: IGLO Method, in *eMagRes*, ed. R. K. Harris and R. L. Wasylishen, 2007.

6) S. F. Boys, F. Bernardi, *Mol. Phys.,* 1970, **19**, 553-566.
